# On the Impact of Biceps Muscle Fatigue in Human Activity Recognition

**DOI:** 10.3390/s21041070

**Published:** 2021-02-04

**Authors:** Mohamed Elshafei, Diego Elias Costa, Emad Shihab

**Affiliations:** Department of Computer Science and Software Engineering, Concordia University, Montreal, QC H3G 1M8, Canada; diego.costa@concordia.ca (D.E.C.); eshihab@cse.concordia.ca (E.S.)

**Keywords:** human activity recognition, wearable sensors, wearable sensor data, machine learning

## Abstract

Nowadays, Human Activity Recognition (HAR) systems, which use wearables and smart systems, are a part of our daily life. Despite the abundance of literature in the area, little is known about the impact of muscle fatigue on these systems’ performance. In this work, we use the biceps concentration curls exercise as an example of a HAR activity to observe the impact of fatigue impact on such systems. Our dataset consists of 3000 biceps concentration curls performed and collected from 20 volunteers aged between 20–35. Our findings indicate that fatigue often occurs in later sets of an exercise and extends the completion time of later sets by up to 31% and decreases muscular endurance by 4.1%. Another finding shows that changes in data patterns are often occurring during fatigue presence, causing seven features to become statistically insignificant. Further findings indicate that fatigue can cause a substantial decrease in performance in both subject-specific and cross-subject models. Finally, we observed that a Feedforward Neural Network (FNN) showed the best performance in both cross-subject and subject-specific models in all our evaluations.

## 1. Introduction

Fatigue is an inevitable consequence when it comes to athletics and incremental exercises [1]. There are a plethora of studies about human fatigue in the literature. Some studies adopt the clinical approach to study human fatigue, where they provide an in-depth definition, characterization, and examination of fatigue. Clinically, fatigue differs based on the type of activities performed by a person. Physical activities generate objective fatigue, which decreases the capability to exert mechanical work [2]. On the other hand, subjective fatigue is generated by intense mental tasks that are characterized by a decline of alertness and mental concentration [3]. These two types of fatigue gradually push the body to reach its limit, breaking the homeostasis state due to the difference between the metabolic energy production/consumption and the accumulation of metabolic waste at the cellular level, conditioning the normal functioning of the organic system [4]. As a result, this can damage the organism, or in more drastic situations, it may lead to overwork, chronic fatigue syndrome, over-training syndrome, and immunity dysfunctions [5].

Recently, several fatigue detection approaches have been proposed in the literature to avoid fatigue-induced injuries. The invasive approach is one of the earliest methods to detect fatigue and requires measuring the lactic acid in the bloodstream to determine the maximal muscle effort that a person can maintain without risking fatigue injuries [6]. The cardio-respiratory approach is another method to detect fatigue however, it is based on a person’s metabolic system. It requires a face mask to measure the circulatory and respiratory systems’ ability to supply oxygen (*O*_2_) to skeletal muscles during sustained physical exercise [7]. Other studies refer to this method as *VO*_2_ max, which stands for the maximum volume of oxygen consumption measured during incremental exercise. Recently, approaches using wearables have been developed to detect fatigue based on the Rating of Perceived Exertion (RPE), a validated subjective measure of fatigue. For example, prior work used wearables to measure fatigue for outdoor running [8].

Despite the abundance of works on fatigue detection in the literature, little is known about its impact on Human Activity Recognition (HAR) systems. Outwardly, fatigue impacts human performance through degradation of exerted force and internally, it impacts heart rate, blood pressure, and core temperature, which can be measured using the appropriate tools. Although previous works (e.g., [9,10,11]) on the impact of fatigue have covered human performance and internal body changes, these works have been focused on clinical approaches that measure levels of lactate, creatine kinase, and *VO*_2_ max. The goal of our work is to study the impact of fatigue on detection models. Fatigue may naturally occur in any human activity, but it poses a bigger challenge for HAR models when identifying physically demanding activities, such as gym activities. For this reason, we focus on studying the biceps concentration curls exercise, which involves flexing one of the most active skeletal muscles at the elbow joint countless times to pick, lift, and pull objects [12,13]. We collect a dataset for biceps concentration curls from 20 volunteers aged between 20–35. Then, we analyze the data patterns that occurred in repetitions during the presence of fatigue and extract all the non-fatigue repetitions into a subset. We extract two sets of features to detect bicep concentration curls, a feature set from the complete dataset and another feature set from the non-fatigue subset. We then contrast these two sets of features to find whether fatigue has affected the number of extracted features. After that, we train five machine learning models to detect biceps concentration curls to observe their performance before and after fatigue presence. In comparison to related work, our contribution in this work is to study the impact of fatigue using the recent wearable approach instead of the clinical ones. Furthermore, we focus on how fatigue impacts the collected data, extracted features, and performance of detection models rather than focusing on human performance and internal body changes as in the clinical approaches.

The rest of the paper is structured as follows, Section 2 demonstrates how we collect our dataset, label its entries, and address related challenges. Section 3 describes our approach to data processing, feature extraction, and experiment setup. In Section 4, we answer four research questions about the impact of fatigue. In Section 5, we discuss other interpretations of our findings. Section 6 concludes our paper.

## 2. Data Collection and Related Challenges

In this section, we present our data collection approach and the challenges involved, i.e., dumbbell suitability and subjectivity of the Rate of Perceived Exertion (RPE). We also discuss the possible solutions to overcome these challenges to provide a high-quality dataset for our study.

### 2.1. Data Collection

Our dataset must contain a sufficient number of biceps concentration curls for two reasons. First, we have to collect enough data entries that capture the impact of fatigue and second, to observe the kinetic changes that occur during the exercise. We use Borg’s scale to evaluating the Rate of Perceived Exertion (RPE) during the exercise, which is a subjective measurement of fatigue within sport science. We explained the Borg’s scale to each volunteer to express their levels of fatigue, as shown in Table 1. Then, we asked 20 volunteers between the ages of 20 to 35 to perform biceps concentration curls using a dumbbell. At scales of 6–10, the volunteer reported no to light feeling of exertion. At scales 11–14, the volunteer reported fairly light to moderate level of exertion. At scales 15 or higher, the volunteer reports a vigorous level of exertion. We believe that RPE is suitable in our case because it is non-invasive, convenient, and user-friendly, making it more practical and less equipment demanding. Moreover, RPE measures peripheral muscle fatigue, given that it provides a more comprehensive view, which includes feedback from cardiovascular, respiratory, and musculoskeletal systems [14]. Furthermore, RPE has proven to model a person’s performance better in the real-world compared to only heart rate monitoring [15] thus, RPE is an appropriate and validated marker of a volunteer’s fatigue [16].

**Figure 1:**
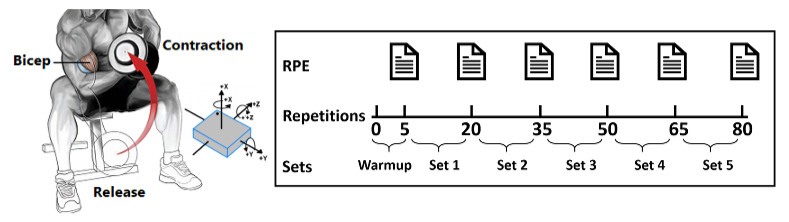
Visualization of data acquisition sessions of biceps concentration curl exercise. Rating of Perceived Exertion (RPE).

We placed a single 50 Hz Neblina Inertial Measurement Unit (IMU) on the volunteer’s wrist during our recording sessions. The IMU device contains a 3-axis accelerometer, a 3-axis gyroscope, and a 3-axis magnetometer. The IMU unit measures one signal for each of the three orthogonal axes per sensor type, resulting in nine signals per IMU. We also placed the Apple Watch Series 4 on the opposite wrist to measure the volunteer’s heart rate. The Apple Watch calculates the number of times the heart beats each minute and supports a range of 30–210 beats per minute. Although Apple Watch uses Photoplethysmography (PPG) for heart rate monitoring, which may inherit inaccuracies, previous work [17] have shown that Apple Watch provided the most accurate readings with no statistical difference compared to Electrocardiography (ECG). Moreover, another work [18] demonstrates that the Apple Watch measures heart rate with clinically acceptable accuracy, and it might be considered safe to use it for cardiac rehabilitation training programs. Therefore, the convenience and accuracy of Apple Watch have motivated us to use the device to measure the volunteer’s heart rate. Once we collect the volunteer’s heart rate and the reported Borg, we multiply the reported Borg rating by 10 to estimate the volunteer’s heart rate during the exercise. This means if both heart rates measured by the Apple Watch and the estimate from the Borg scale are similar, we have a higher confidence on the validity of reported RPE for each volunteer. In the rare case of dissimilarity between the Borg scale and the measured heart rates, we average the reported RPE with the measured heart rate converted to RPE, as similarly done in previous work [19]. Figure 1 shows a visual illustration of the biceps concentration curl exercise where we demonstrate the exercise to each volunteer as the following:

1. The volunteer should sit down on a flat bench with one dumbbell placed between their legs;
2. The volunteer should be in a release position by using their right arm to pick the dumbbell up. Then, place the back of their right upper arm on the top of the inner right thigh. The volunteer should rotate the palm of their hand until it is facing forward away from the thigh. Once, their arm is extended with the dumbbell above the floor then; the volunteer is in the correct release position, as shown in Figure 1;
3. While the volunteer is holding the left arm stationary, they curls the weights forward while contracting the biceps as they breathe out. With the forearms movement only, the volunteer continues until their biceps are fully contracted, and the dumbbells are at shoulder level. The volunteer should hold the contracted position for a second, as shown in Figure 1;
4. The volunteer should slowly begin to bring the dumbbells back to the release position as their breathe in. Avoid swinging motions at any time;
5. Repeat for 15 repetitions. Then repeat the exercise the left arm to carry the dumbbell;
6. The volunteers are equally allowed to rest for 2 min between sets, which is a common exercise protocol in previous works [20,21].

In the end, each volunteer completed 5 warm-up repetitions followed by 15 repetitions per set for a total of 5 sets per hand, as shown in Figure 1. The volunteers reported their RPE after each set, including the warm-up, yielding 6 RPE values per hand. In total, we collected longitudinal data for 3000 concentration curl repetitions.

### 2.2. Data Collection Challenges

We encountered two major challenges during our data collection procedure: Dumbbell suitability and RPE subjectivity. We presumed that selecting a certain dumbbell weight for collecting data from different volunteers may induce a loose variance in their fatigue measures, such as the number of repetitions or completion time per set. In addition, using subjective measures, such as RPE, we could introduce a dependency between the correctness of the selected Borg rating and the volunteers’ body awareness.

The first challenge we encountered is the suitability of the dumbbell. The process of collecting data from 20 volunteers raises the issue that not all volunteers can equally perform the exercise. To address this challenge, we provided all volunteers with three groups of dumbbells: Light-weight which includes 1.1 kg and 2.2 kg dumbbells, medium-weight which includes 4.5 kg dumbbells, and heavy-weight which includes 9 kg dumbbells, as shown in Table 2. Then, we asked each volunteer to perform at least 2 sets of bicep concentration curl repetitions until they felt fatigued. As expected, when volunteers used light-weight dumbbells, they were able to perform a high number of repetitions per set but fewer sets in total (see row 1 and 2 in Table 2). This resulted in long recording sessions with a lot of similar data entries until volunteers reached fatigue. On the other hand, when we look at the results obtained with a heavy-weight dumbbell (9 kg), volunteers were able to accomplish fewer repetitions and fewer sets in total (see row 4 of Table 2). This resulted in short recording sessions with fewer data entries however, momentum changes were not captured clearly throughout the exercise because volunteers reached fatigue quickly. We found the results obtained with medium-weight dumbbells (4.5 kg) to be the best compromise between the recording time length and the momentum changes as volunteers reached fatigue more gradually. Volunteers were able to perform 16 repetitions per set, which each set taking on average 1 min and 17 s to complete (see row 3 in Table 2). In addition, previous work found that a similar dumbbell weight (4.5 kg) provided the best trade-off between recording sessions length and the occurrence of fatigue on exercises [22].

The second challenge we encountered is the subjectivity of RPE, where volunteers may rate their exertion level differently based on their feeling of exhaustion. This issue may lead them to inaccurately report their levels of exertion throughout the exercises. The third challenge is the familiarity with RPE: Some volunteers were not familiar with the RPE before the data acquisition sessions. This may lead them to rate their exertion level incorrectly, even worsening the effects of the previous challenge if not appropriately addressed. To address both of these challenges, we apply a min-max normalization to the RPE value based on the current set to account for subjective differences in RPE. For illustration, we set the minimum value based on the RPE reported after the warm-up, which often ranges from 10 to 12. Then, we set the maximum value to the highest RPE on the Borg scale, which is 20. We use such a fixed value as the maximum RPE because if we use the values reported from the set, it will cause the current label to depend on future data, which is not methodologically sound. The longitudinal nature of the data acquisition sessions also helped participants to become more familiar with the scale as they performed more sets. Therefore their use of the RPE potentially evolved across consecutive sets.

## 3. Data Processing and Experiment Setup

In this section, we describe how we prepared our dataset through data processing and feature extraction. Then, we describe how we set up our experiment to evaluate the impact of fatigue in HAR models.

### 3.1. Data Processing and Feature Extraction

Our collected data consists of an accelerometer, gyroscope, and magnetometer readings, where each reading is composed of the three orthogonal axes from each sensor type. These signals were converted to the three-dimensional Cartesian coordinates (x, y, z) for better representation. At the end of this conversion, our data contains a total of nine signals: The 3-axis of accelerometer, gyroscope, and magnetometer.

**Figure 2:**
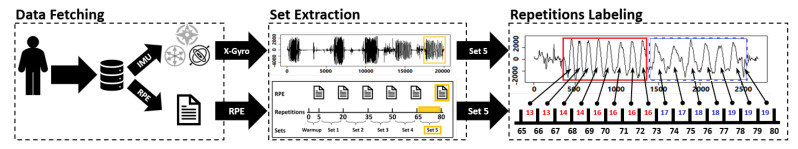
A visual example of extracting and labeling repetitions of the fifth set from the gyroscope’s *x*-axis.

**Figure 3:**
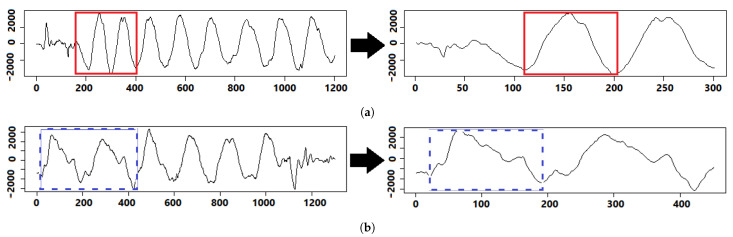
Raw bicep repetitions data collected from the *x*-axis of the gyroscope. Red thick boxes highlight repetitions without fatigue, while blue dashed boxes highlight repetitions with fatigue. (**a**) Extracting non-fatigue bicep concentration curl repetitions from the fifth set. (**b**) Extracting fatigue bicep concentration curl repetitions from the fifth set.

We illustrate in Figure 2, how we extracted and labeled repetitions for each volunteer. In the figure, we exemplify the three main steps to extract and label repetitions, by using an example of a fifth set from the gyroscope’s x-axis. The first step is the data fetching, where we fetch volunteer’s data, including their IMU and RPE. In this example, we chose the gyroscope’s x-axis because it best visualizes the repetitions however, we applied the same process for all 3-axis of accelerometer, gyroscope, and magnetometer. The second step is the set extraction, where we extract all five sets of concentration curls along with their corresponding RPE values reported by the volunteer. We illustrate the extraction of the fifth set because it often has the highest ratio of fatigue and non-fatigue repetitions. The third step is the repetition labeling, where we look closely into all exercise sets to find two distinct groups of non-fatigue and fatigue repetitions. The non-fatigue repetitions are highlighted by the red thick border, while the blue dashed border highlights the fatigue ones. The troughs indicate that the volunteer has reached the release position, while peaks indicate that they have reached the contraction position, as demonstrated in Section 2.1. A recent work on quantifying muscle fatigue has selected an RPE value of 16 as the threshold of true fatigue to estimate the declines in muscle strength during tasks [23]. Therefore, we extract and label each repetition manually according to the RPE values reported for the set, where we label repetitions with reported RPE values larger than 16 as fatigue. Figure 3a shows a zoomed-in look on a group of eight non-fatigue repetitions from the fifth set, where each non-fatigue repetition can be clearly extracted between two troughs. Similarly, we extract a complete fatigue repetition between two troughs, as shown in Figure 3b. We observe that a non-fatigue repeat is relatively symmetrical on the other hand, the fatigue repeats have a relatively positive skew, as shown in Figure 3. We repeat the same process for all sets. The used IMU has a synchronized 3-axis accelerometer, gyroscope, and magnetometer, which allows us to use the same timestamps from the gyroscope’s x-axis to extract and label repetitions for all the remaining signals (other axes of gyroscope, accelerometer, and magnetometer). After we processed all data from 20 volunteers, we were able to extract a total of 3000 repetitions recorded from nine time-series signals with one IMUs.

**Figure 4:**
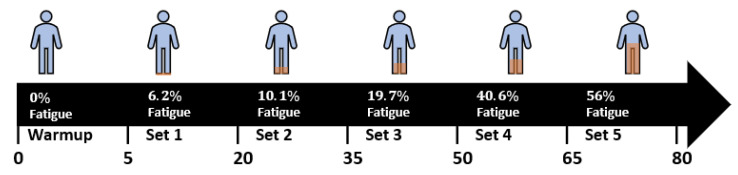
A visualization of the weighted average of fatigue shares per exercise sets in the collected data.

To have a better understanding of the prevalence of fatigue in our dataset, we quantify the fatigue repetitions across the five sets. Then, we calculate the average of fatigue repetition share per volunteer, as shown in Figure 4. The volunteers did not report any fatigue repetitions at the warmup set thus, the share of fatigue for this set is 0%. However, in the first set, 19 volunteers reported the last repetition as fatigue, which represents 1 out of 15 repetitions (6.6%), while 1 volunteer reported no fatigue (0%). Hence, the average share of fatigue repetitions in the first set was reported at 6.2%. As we see in Figure 4, the average share of fatigue repetitions increases at each subsequent set as volunteers become more progressively tired, reaching an average of 56.0% of fatigue repetitions per volunteer in the fifth set.

After we extract all repetitions individually, we extract 27 features by computing the mean, Mean Absolute Deviation (MAD), and Standard Deviation (SD) for all nine signals. We also computed three extra signals from the gyroscope: Roll, pitch, and yaw by integrating angular velocity over time to give us the movement angle. Then, we extract nine extra features from these signals so that we have a total of 36 extracted features. We select these features primarily because they have been commonly used in previous works to detect human activities and have shown to yield good performance [8,24,25]. In addition, previous work have reported that these features perform well in displaying signal changes over a span of time during an activity to spot possible anomalies [26]. After this entire feature engineering step, we were able to extract 36 features from 3000 repetitions recorded from nine time-series signals.

### 3.2. Experiment Setup

**Figure 5:**
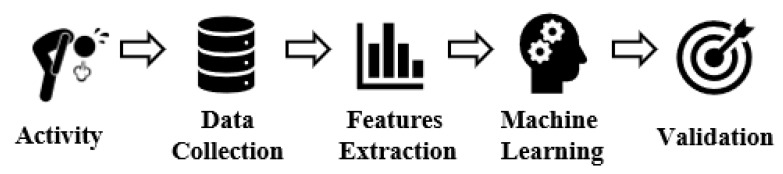
Overview of the wearable approach based human activity recognition system.

In this work, we study fatigue’s direct and indirect impact on HAR systems: The changes that the presence of fatigue caused on the raw collected data, the impact of fatigue on the significant features, and the performance of machine learning models in a dataset with the presence of fatigue. In Figure 5, we show an overview of the methodology used in this study. We select biceps concentration curls as an activity for this work and collected our dataset from 20 volunteers using an IMU attached to their wrists. We manually labeled each repetition in our dataset as containing fatigue or not containing fatigue. To study the impact of including fatigue in our dataset, we group all the non-fatigue repetitions into a subset. Then, we extract two sets of features, a feature set from the complete dataset and another feature set from the non-fatigue subset. After that, we compare the two sets of extracted features to find whether fatigue affects the number of significant features in detecting biceps concentration curls. Next, we train five machine learning models to detect biceps concentration curls to observe their performance before and after the presence of fatigue. The goal of this step is to evaluate how fatigue may affect the models when recognizing human-activity.

Machine learning classification models are often reported in comparative works with high-performance rates in detecting human activities using wearable IMU on the wrist [27,28,29]. So given the fact that these models were effective in detecting human activity using similar data to what we collected, we select five of these models to detect bicep concentration curl repetitions [30]. The first model is the Generalized Linear Models (GLM) which has been adopted to analyze and count the number of walking steps in a previous study [31]. The second model is the Logistic Regression (LR) which has been used to analyze and detect human activities [32]. The third model is Random Forest (RF), which has been used to detect and classify human actions using wearable motion sensor networks [33]. The fourth model is the Decision Trees (DT) which has been used to count and classify ambulatory activities using eight plantar pressure sensors within smart shoes in a previous study [34]. The fifth model is the Feedforward Neural Network (FNN) which has been used to detect and count repetitions for complex physical exercises [35]. Furthermore, we took into consideration two approaches for each model, the subject-specific and cross-subject approaches:

- Subject-specific model: The model is trained to fit an individual or individuals with the same fatigue response pattern, however, it requires previous data from each subject;
- Cross-subject model: The model is trained to fit a group of individuals and utilize their previous data to fit new users.

A cross-subject model is optimized for working with a large number of users, which is more realistic in real-world applications. On the other hand, a subject-specific model that is tailored to individual data tends to outperform the cross-subject model.

## 4. Experiment Evaluation

Our main goal in this section is to study the impact of fatigue on the collected data, the number of significant features, and the models’ performance. In addition, we evaluate the impact of fatigue on subject-specific and cross-subject models. Specifically, we address the following research questions:

- RQ1: How does fatigue impact the collected data?
- RQ2: What impact can fatigue impose on the extracted features?
- RQ3: What is the impact of fatigue on subject-specific biceps repetitions models?
- RQ4: What is the impact of fatigue on cross-subject biceps repetitions models?

Next, we detail the motivation, approach, and the findings for each research question.

### 4.1. RQ1: How Does Fatigue Impact the Collected Data?

Motivation: We believe that fatigue impacts the collected data by changing its patterns, leading to a snowball effect, affecting the extracted features and HAR models’ performance. Hence, we want first to capture the data pattern changes, which might occur during the data collection process.

**Figure 6:**
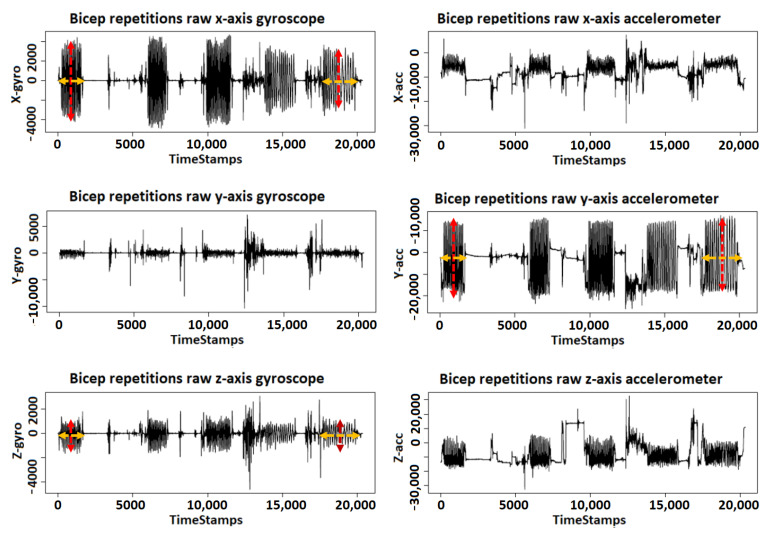
A visualization of the impact of fatigue on collected data.

Approach: To address this research question, we look into the data provided by the IMU that contains the 3-axis gyroscope and accelerometer. We excluded the magnetometer for simplicity as it did not show any significant changes in the magnetic field regarding direction or strength during the exercise. We started with a visualization of the impact of fatigue on collected data to evaluate the data pattern changes. Figure 6 shows an example of the five sets of biceps repetitions using the gyroscope and the accelerometer signals. The *X*-axis represents the vertical displacement, which is the distance between the highest and lowest positions of the volunteer’s hand during bicep extension and flexion. The *Y*-axis represents the horizontal displacement, which is the sideways vibration of the volunteer’s hand during bicep extension and flexion. The *Z*-axis represents the depth displacement, which is the farthest and nearest positions of the volunteer’s hand from their body during bicep extension and flexion. We select the *X*-axis from the gyroscope and *Y*-axis from the accelerometer because they provide the best visualization for the angular velocity and sideways vibration of the volunteer’s hand. Similarly, we showcase the *Z*-axis from the gyroscope as it provides the best visualization of the farthest and nearest positions of the volunteer’s hand. We use the first set of bicep repetition as a reference set to comparatively measure the data pattern changes. The rationale behind this is that the first set usually contains the least number of fatigue repetitions. Therefore, as the fatigue accumulates in later sets, we would be able to differentiate the changes in the data patterns. The first set also always contains 15 biceps repetitions for all of the 20 volunteers.

We look for data pattern changes along the horizontal axis which indicates the changes in completion time whereas the vertical axis indicates the changes in angular velocity according to muscular endurance [36]. To analyze the data pattern changes in the horizontal axis, we measured the time required to complete the first set of biceps repetitions for each volunteer, which is the time interval from the 1st repetition until the end of the 15th repetition. We repeated the same approach to measure the completion time of the remaining sets separately. Then, we calculate the difference in completion time between each set compared to the first set. To analyze the data pattern changes in the vertical axis, we measured the absolute magnitude of each repetition in the first set to calculate the muscular endurance [36] during the first set of biceps repetitions. We repeated the same approach to measure the muscular endurance for the remaining sets separately. Then, we calculate the drop in muscular endurance between each set compared to the first set.

Result: Table 3 shows the increase in completion time for each set in relation to the first set. In the 2nd set, the average increase in completion time is 1.7%, which is considerably a small change to the 1st set. The reason is that the 2nd set is usually the introductory stage of fatigue, where fatigue occurs for the first time at the last 1 or 2 repetitions. When we look at the 3rd set, we found the average increase in completion time to have increased to 8.1%. At the 4th set, volunteers take on average 14.3% more time to finish their exercises, compared to the time they took in the 1st set. Comparing the 4th set to the 3rd set, the 4th set contains almost twice the number of fatigue repetitions than the 3rd set, resulting on substantial increase in the time to complete metric, from 8% to 14%. At the 5th set, we found that the average increase in completion time is 31.0%, more than twice the increase observed in the 4th set. The reason is that the 5th set contains at least eight fatigue repetitions, which indicates that fatigue impacts later sets to a much larger extent, slowing down bicep movements and increasing the time completion for the set. As a result, fixed-size and non-overlapping windows will no longer be suitable to capture full repetitions because of its narrow fit, especially, at the 4th and 5th sets.

In Table 4, we present the changes of muscular endurance for each of the five sets as the fatigue accumulates during repetitions in the later sets. It is possible to measure the muscular endurance using the angular velocity from the gyroscope [36] rather than using the accelerometer. Therefore, we observe that fatigue decreases the muscular endurance according to the *X*- and *Z*-axes from the gyroscope by an average of −2.4% and −3.9%, respectively. However, we do not observe a substantial decrease on the muscular endurance using the *Y*-axis from the accelerometer, with a small average change of only 0.4%. Overall, we observe that the average muscular endurance decreases in the later sets as the fatigue accumulates in the repetitions. For example, the 2nd and 3rd sets maintain a muscular endurance similar to the compared 1st set. However, the muscular endurance decreases by an average of 5.5% in the 4th set, and 4.1% in the 5th set, as fatigue accumulates over time. This could negatively impact data filtering, especially in the case of peak filtering, because such a filter may exclude a complete bicep repetition if it did not meet the peak threshold, especially, at the 4th and 5th sets.

### 4.2. RQ2: What Impact Can Fatigue Impose on the Extracted Features?

Motivation: We believe that if fatigue affects the collected data, it may affect the extracted features from the same data. In other words, some features may appear to be significant in detecting biceps repetitions without fatigue, but become less significant at later sets, where fatigue often occurs. We think that a factor such as fatigue can deform the patterns of these features reducing their correlation hence, some extracted features may be more sensitive to fatigue than others.

Approach: To address this research question, we have to analyze the effects of fatigue on the extracted features. To that aim, we extract three main features from our complete dataset and contrast them with the features extracted in the non-fatigue subset. Our goal is to investigate whether fatigue could influence the number of significant features of a HAR model. The three main features are mean, MAD, and SD. Our complete dataset and the non-fatigue subset contain 12 data signals where 9 data signals are collected from the 3-axes of gyroscope, accelerometer, and magnetometer; 3 data signals represent the rotations on *X*-, *Y*-, and *Z*-axes which are roll, pitch, and yaw. In total, we have 36 extracted features in our complete dataset and the non-fatigue subset. We use Spearman’s rank correlation coefficient with a significance allowance of 0.1 to show how these extracted features correlate with bicep repetitions [37].

**Figure 7:**
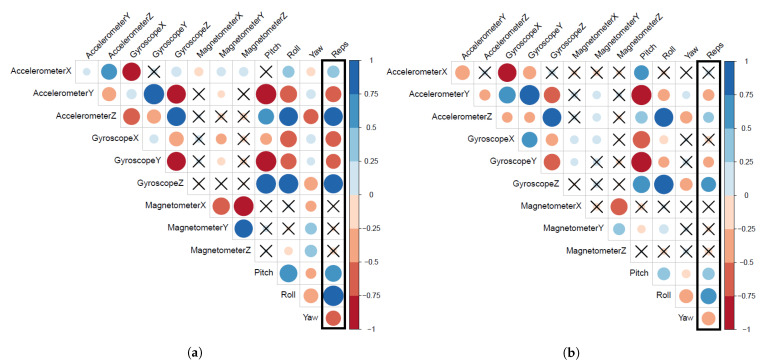
Graphical display of the differences in the correlation matrices of the 12 extracted features (mean) and the bicep repetitions with and without fatigue. (**a**) Correlation matrix of the 12 mean features and the bicep repetitions in the non-fatigue subset. (**b**) Correlation matrix of the 12 mean features and the bicep repetitions in our complete dataset.

Figure 7 presents the correlation matrix for 12 mean features and the bicep repetitions. The positive correlations are displayed in blue and negative correlations are presented in red. The color intensity and the size of the circle are also proportional to the correlation coefficients whereas, the insignificant correlations are marked with ×. Figure 7a shows that bicep repetitions have significant correlations with 9 out of 12 mean features extracted from the non-fatigue subset. These 9 significant features are (X,Y,Z)-Accelerometer, (X,Y,Z)-Gyroscope, pitch, roll, and yaw. On the other hand, Figure 7b shows that bicep repetitions have significant correlations with 7 out of 12 mean features extracted from our complete dataset where fatigue exists during the exercise. These 7 significant features are (Y,Z)-accelerometer, (Y,Z)-gyroscope, pitch, roll, and yaw. We can observe two impacts of fatigue on extracted features. First, some mean features correlations became insignificant to bicep repetitions such as X-accelerometer and X-gyroscope. Second, an overall drop in the correlation coefficient values for all mean features, as presented by the faint color intensity and the shrink of circle sizes.

**Figure 8:**
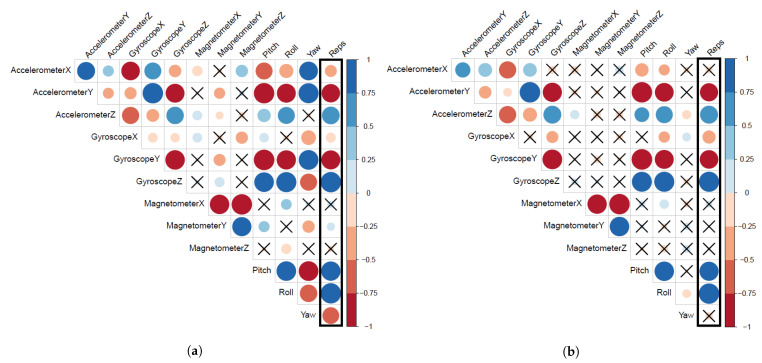
Graphical display of the differences in the correlation matrices of the 12 extracted features (MAD: Mean Absolute Deviation) and the bicep repetitions with and without fatigue. (**a**) Correlation matrix of the 12 MAD features and the bicep repetitions in the non-fatigue subset. (**b**) Correlation matrix of the 12 MAD features and the bicep repetitions in our complete dataset.

To strengthen the evidence that points to fatigue as the potential cause of these impacts, we believe that similar observations should exist for MAD and SD features. Figure 8 presents the correlation matrix for 12 MAD features and the bicep repetitions in our complete dataset and the non-fatigue subset. Figure 8a shows that bicep repetitions have significant correlations with 10 out of 12 MAD features extracted from the non-fatigue subset. On the other hand, Figure 8b shows that bicep repetitions have significant correlations with seven out of 12 MAD features extracted from our complete dataset. Again, we encounter a similar effect to the aforementioned ones in extracted features (mean). Some MAD features correlations became insignificant to bicep repetitions such as X-accelerometer, Y-magnetometer, and yaw. However, we did not observe a major drop in all MAD features’ correlation coefficient values, only the newly three mentioned insignificant suffered from a drop in the correlation coefficient values.

**Figure 9:**
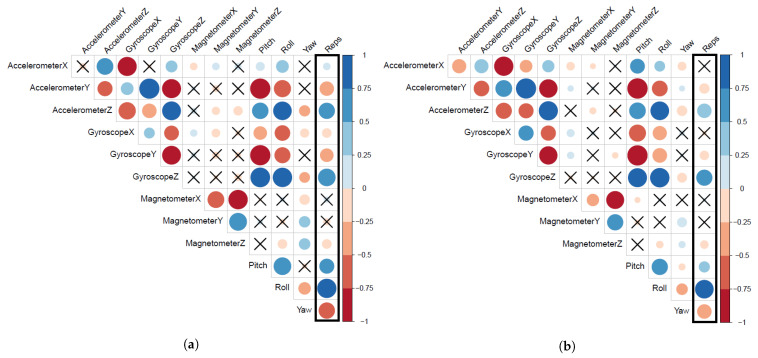
Graphical display of the differences in the correlation matrices of the 12 extracted features (SD: Standard Deviation) and the bicep repetitions with and without fatigue. (**a**) Correlation matrix of the 12 SD features and bicep repetitions in the non-fatigue subset. (**b**) Correlation matrix of the 12 SD features and the bicep repetitions in our complete dataset.

Figure 9 presents the correlation matrix for 12 SD features and the biceps repetitions in our complete dataset and the non-fatigue subset. Figure 9a shows that bicep repetitions have significant correlations with 10 out of 12 SD features extracted from the non-fatigue subset. On the other hand, Figure 9b shows that bicep repetitions have significant correlations with eight out of 12 SD features extracted from our complete dataset. Once more, some SD features correlations became insignificant to bicep repetitions such as the *X*-axis for both accelerometer and gyroscope. We also observe a slight drop in the correlation coefficient values for all SD features, as presented by the faint color intensity and the shrink of circle sizes. At this point, we clearly observe the same recurring effects when fatigue is introduced to the data, which indicates that fatigue impacts the significant features of a HAR model.

Table 5 shows the features extracted from each dataset. In RQ1, we pointed out that the *X*-axis represents the vertical displacement, which has the largest angle of movement and linear acceleration. However, fatigue often affects acceleration greatly compared to the angle of movement due to the movement nature of the biceps muscle [38]. Therefore, changes on the linear acceleration, measured by the accelerometer, affected the significance of its extract features. On the other hand, the angular velocity, measured by the gyroscope, remains relatively steady because of the fixed angle of movement of bicep muscles. Regarding the low number of significant features from the magnetometer, we believe that fatigue did not impact that feature. But mostly, the magnetometer is not an optimal sensor as it did not reveal any significant characteristics readings about the magnetic field’s direction or strength, as previously mentioned in RQ1. Most of the features extracted from the magnetometer were insignificant.

Result: Our findings show that fatigue significantly impacts the extracted features, by hindering their correlation coefficient values to the extent of turning some significant features into insignificant ones. We were able to extract 9 mean, 10 MAD, and 10 SD features from the none-fatigue subset for a total of 29 significant features with a significance allowance of 0.1. However, once the fatigue was introduced in the data, we were only able to extract 7 mean, 7 MAD, and 8 SD features for a total of 22 significant features, as shown in Table 5. This indicates that fatigue, once introduced in the dataset, reduced the significance of 7 features (24% of total significant features).

### 4.3. RQ3: What Is the Impact of Fatigue on Subject-Specific Biceps Repetitions Models?

Motivation: Given that fatigue impacted the extracted features, we want to investigate how it could affect the biceps detection models’ performance. The presence of fatigue has the potential to decrease the model’s performance in recognizing human activities. Therefore, we start with subject-specific models to examine their ability to detect biceps concentration curl in a dataset with a progressive inclusion of fatigue repetitions.

Approach: To address this research question, we use the five detection models mentioned in Section 3.2. These models use the 22 significant features extracted from our complete dataset to eliminate weak features that turn to insignificant once fatigue occurs. We use these models to detect biceps repetitions in our dataset. Then, we calculate the accuracy using the confusion matrix shown in Table 6, where non-repetition represents an incomplete repetition or any random movement, and repetition represents a completed repetition, whether it contains fatigue or not. We perform six 10-fold cross-validation runs using the non-fatigue subset where we replace 10% of the non-fatigue repetitions in the subset with fatigue repetitions from our complete dataset per experiment. Note that all models are trained and tested with similar levels of fatigue to simulate a realistic use-case. Since fatigue is a natural consequence of any physical activity, it is expected to occur in the training data of models as well as when a final users use wearable devices for HAR. These runs are done over all participants, and then averaged, as shown in Table 7. This allows us to observe the effects of fatigue on the detection models gradually, as more fatigue repetition is added to the dataset. We use the first 10-fold cross-validation run as a reference point because there are no fatigue repetitions in the dataset to hinder models’ performance. Then, we calculate the accuracy (1), precision (2), recall (3), and F1 (4) for each run. Table 7 shows the performance averages for the six 10-fold cross-validation runs over all participants per model. Each △ F1* row shows the difference in the model’s performance, compared to the performance obtained in the first run (no fatigue).

(1) $$Accuracy=\frac{True(Repeat+NonRepeat)}{True(Repeat+NonRepeat)+False(Repeat+NonRepeat)}$$

(2) $$Precision=\frac{True(Repeat)}{True(Repeat)+False(Repeat)}$$

(3) $$Recall=\frac{True(Repeat)}{True(Repeat)+False(NonRepeat)}$$

(4) $$F1=\frac{2*Precision*Recall}{Precision+Recall}$$

Result: Our findings show that the more fatigue added to the dataset, the steeper the decline in performance of the five models. In fact, Table 7 shows that replacing as little as 10% of the repetitions with fatigue repetitions can drop the GLM models’ performance by 8%, and 11% for DT. If we replace an additional 10% of the repetitions with fatigue repetitions, all models’ performance decrease by at least 21% (FNN and DT). The decrease in the performance can be as severe as 30% in the RF model. Such findings indicate that, for some models (e.g., GLM, LR, and RF), it only takes 20% of fatigued repetitions to decrease a model’s performance by more than 20%. The impact in the model’s performance is even more significant when we reach to 40% and 50% of fatigue repetitions. With half the repetitions containing fatigue, the models lose between 47% (DT) to 57% (GLM) of its original performance, which may compromise the reliability of HAR systems that do not take fatigue properly into account.

### 4.4. RQ4: What Is the Impact of Fatigue on Cross-Subject Biceps Repetitions Models?

Motivation: We observed that the impact of fatigue on subject-specific models were significant, hindering the performance of models by at least 20% when fatigue is present in a quarter of all repetitions. We believe cross-subject models may be similarly affected, perhaps even to a greater extent, given that these models tend to underperform the subject-specific models. Hence, we assess the impact of fatigue on the generality of the models and the ability to detect biceps repetitions across different subjects.

**Figure 10:**
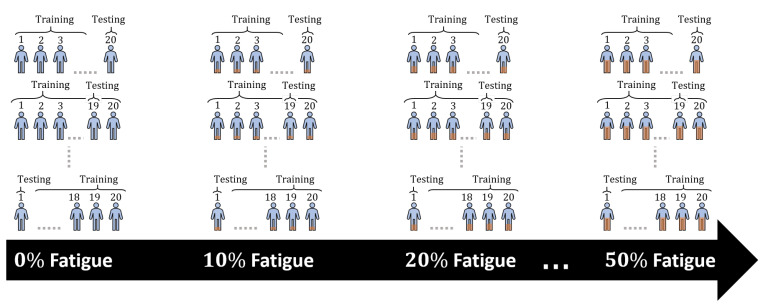
A partial representation of the six Leave-One-Out Cross-Validations (LOOCV) with K=20 and different percentages of fatigue.

Approach: Our approach used to answer RQ4 is similar to RQ3. We use the 22 significant features extracted from our complete dataset and use the five detection models aforementioned in Section 3.2. We perform six Leave-One-Out Cross-Validations (LOOCV) runs where K equals the number of volunteers, K = 20. Figure 10 shows a partial representation of the six LOOCV runs with K = 20 for using the data of 20 volunteers individually. Each LOOCV run consists of 20 iterations, in the 1st iteration, we use the data from 19 volunteers to train our models then use the 20th volunteer’s data to test the models. At last, in the 20th iteration, we should have used all volunteers data for testing except the 1st volunteer therefore, we train the model using all the 19 volunteers dataset then, used the 1st volunteer’s data for testing. We calculate the precision, recall, accuracy, and F1-score per iteration then report the averages. We repeat the LOOCV run after we replace 10% of each volunteer’s data with fatigue repetitions from each individuals data, recursively. We use the first LOOCV run as a reference point because there are no fatigue repetitions in the individuals data to affect the models’ performance. Similarly to the approach used in RQ3, all models are trained and tested with the same share of fatigue repetitions, to simulate a realistic use-case. Then, we calculate the accuracy (1), precision (2), recall (3), and F1 (4) for each run. Table 8 shows the performance averages for the six LOOCV runs per model. Each △ F1* rows show the comparison of the model’s performance against the performance obtained in the first run (no fatigue repetitions).

Result: Similar to RQ3, our finding indicates fatigue significantly impacts the performance in all five models. Table 8 shows that replacing as little as 10% of the repetitions with fatigued ones can drop a model’s performance by 6% for RF, and down to 13% for GLM and LR. If we replace an additional 10% of the repetitions with fatigued ones, the models’ performance decrease by 20% for FNN and DT, and down to 25% for LR. Once the fatigue reaches 30% of repetitions, we see a sharp decrease in all models by at least 30%. This trend continues, as once the fatigue repetitions reach 50% of the dataset, the HAR models’ performance decrease by at least 41%. We observe a negative linear effect in some models’ performance as the fatigue increases. For instance, the performance of DT models decreases by an average of 10% for every 10% increase of fatigue in the dataset.

## 5. Discussion

In this section, we discuss the findings from our four research questions. In RQ1, our finding shows that fatigue can lead to changes in data patterns over time. A similar finding to ours is shown in a previous work [39], which suggests a decrease in the mean power frequency of the accelerometer readings trend with increasing biceps muscle fatigue. The authors also reported that accelerometers should be used to sense skeletal muscle vibrations, which can reduce the error of estimating fatigue up to 50%. Therefore, we adopted a similar approach in RQ1, where we use a time series dataset collected using an inertial sensor that includes accelerometers to observe data pattern changes along the horizontal and vertical axes. In other words, this allows us to find a correlation between fatigue and data pattern changes that occur horizontally related to completion time and vertically associated with the muscular endurance and angular velocity.

In RQ2, we investigated the effects of the data pattern changes, associated with fatigue, in the feature extraction outcome. Previous work [40] shows that muscle fatigue affects the Electromyography (EMG) data signals collected from biceps by increasing the Root Mean Square Error (RMSEs) of extracted features. Similarly, our findings show that fatigue can hinder the correlation values of some of the extracted features to the extent of turning them into insignificant features. However, if we look at this problem from another perspective, we can label the extracted features as fatigue-resistant. Meaning, although fatigue existed in the dataset, these features remain significant. As a result, we can develop a group of fatigue-resistant features that can counter data pattern changes due to fatigue and remain valuable to detect bicep activity such as biceps concentration curls. It is important to mention, however, that these features are still affected by fatigue as their correlation coefficient values drop, as previously mentioned in RQ2.

In RQ3, our findings show that the more fatigue is added to the dataset, the steeper the decline in performance is on the five subject-specific models. Our findings show that the impact of fatigue can indeed disrupt the models’ performance if not taken properly into account. From the evaluated models, our results indicate that FNN outperforms all other models in terms of precision, recall, accuracy, and F1-score in most cases. We did expect the highest performance from FNN compared to other models as this occurred in previous studies [41,42,43,44]. These studies show that neural networks have significantly better pattern recognition compared to other machine learning models especially, when it comes to periodic activities where extracted features inherit periodicity. Moreover, a popular reason for FNN performance supremacy is its robustness against small-to-moderate changes in the data. Other models, such as DT, has shown to be less robust to fatigue, as even smaller data pattern changes can cause a large change in the structure of the tree.

In RQ3 and RQ4 we compare the performances of the subject-specific and the cross-subject models. We observe a similar and significant performance loss in both models, with a loss of more than 20% if the dataset contains 20% or more of fatigue repetitions. Once again, the FNN has shown to be the most robust of the five evaluated models. This result is corroborated by another related work [45], which reported that FNN maintained the highest rate of accuracy in cross-subject experiments, when detecting fatigue in volunteers driving their vehicles.

To further examine whether our approach adequately accounts for the impact of fatigue, we repeat RQ3 and RQ4 using all features. We use all the 36 extracted features, including the seven features that became insignificant due to the presence of fatigue. This allows us to observe the performance of detection models using all features versus models using only fatigue-resistant features. According to Table 9 and Table 10, fatigue impacts a model’s performance to an even greater extent compared to the models based on 22 fatigue-resistant features presented in RQ3 and RQ4. This result corroborates with our previous analyses, showing that the extraneous features are unlikely to contribute to detecting biceps concentration curls when fatigue is present. It is important to highlight, however, that models using all features do outperform the fatigue-resistant models when the presence of fatigue is very low in the dataset (no fatigue or fatigue data at 10%).

## 6. Conclusions

This paper introduced the impact of fatigue on HAR models for biceps concentration curls as an interesting and impactful data science problem. Specifically, its significant challenges arise from analyzing the IMU collected data, selecting the suitable features, and evaluating the performance of HAR models in a dataset with realistic levels of fatigue. Throughout our study, we found that fatigue often occurs in later sets extending duration time up to 31% compared to the first set and decreasing the muscular endurance down to 4.1%. This leads to a change in data patterns, which causes a series of impacts such as hindering extracted features thus, decreasing models’ performance. As a result, the higher the presence of fatigue in the dataset, the steeper the decline in all model performance. Our findings also showed that FNN maintained the highest performance for cross-subject and subject-specific validations, respectively. Our results indicate that fatigue was a serious problem for machine learning models and we advise practitioners to take fatigue into consideration to develop and deploy accurate HAR systems. The results presented in this work are useful and represent a solid start for enhancing real-world applications for HAR and overcoming the inevitable impact of fatigue.

## Figures and Tables

**Table 1:** Borg G.A. psychophysical bases of perceived exertion [16].

Perceived Exertion	Borg Rating	Examples
None	6	Reading a book, watching television
Very, very light	7 to 8	Tying shoes
Very light	9 to 10	Chores like folding clothes that seem to take little effort
Fairly light	11 to 12	Walking through the grocery store (without speeding up your breathing)
Somewhat hard	13 to 14	Brisk walking (moderate effort and speeding up your breathing)
Hard	15 to 16	Bicycling, swimming, (vigorous effort and get the heart pounding)
Very hard	17 to 18	The highest level of activity you can sustain
Very, very hard	19 to 20	A finishing kick in a race or activity that you can not maintain for long

**Table 2:** The nominated dumbbells weights for the data collection process, the values reported are averages.

Weight (∼kg)	Repetitions	Sets	Repetitions/Set	Completion Time
1.1 kg	2960	60	42.0	2 Min, 30 S
2.2 kg	2417	79	31.0	1 Min, 45 S
4.5 kg	1580	100	16.0	1 Min, 17 S
9 kg	860	60	9.0	1 Min, 10 S

**Table 3:** The increase in the time to complete a set compared to the 1st set.

Axis-Sensor	2nd Set	3rd Set	4th Set	5th Set	Avg.
X-Gyroscope	+2.0%	+6.0%	+17.0%	+33.0%	+14.5%
Z-Gyroscope	+1.7%	+11.0%	+15.0%	+45.0%	+18.2%
Y-Accelerometer	+1.5%	+7.4%	+11.0%	+15.0%	+8.7%
Avg./set	+1.7%	+8.1%	+14.3%	+31.0%	

**Table 4:** The change in muscular endurance represented in vertical shrinks, compared to the 1st set.

Axis-Sensor	2nd Set	3rd Set	4th Set	5th Set	Avg.
X-Gyroscope	+0.7%	+1.2%	−6.3%	−5.2%	−2.4%
Z-Gyroscope	+0.5%	+1.7%	−10.4%	−7.5%	−3.9%
Y-Accelerometer	+0.6%	+0.3%	+0.3%	+0.4%	0.4%
Avg./set	+0.6%	+1.1%	−5.5%	−4.1%	

**Table 5:** Table of the significant (✓) and insignificant (×) features extracted from both the non-fatigue subset and complete dataset; the changed features are in highlighted bold.

			Non-Fatigue Subset			Complete Dataset		
			Mean	MAD	SD	Mean	MAD	SD
**Sensors and Axes**	**Acc.**	*X*-axis	**✓**	**✓**	**✓**	**×**	**×**	**×**
		*Y*-axis	✓	✓	✓	✓	✓	✓
		*Z*-axis	✓	✓	✓	✓	✓	✓
	**Mag.**	*X*-axis	×	×	×	×	×	×
		*Y*-axis	×	**✓**	×	×	**×**	×
		*Z*-axis	×	×	✓	×	×	✓
	**Gyro.**	*X*-axis	**✓**	✓	**✓**	**×**	✓	**×**
		*Y*-axis	✓	✓	✓	✓	✓	✓
		*Z*-axis	✓	✓	✓	✓	✓	✓
		Roll	✓	✓	✓	✓	✓	✓
		Pitch	✓	✓	✓	✓	✓	✓
		Yaw	✓	**✓**	✓	✓	**×**	✓

**Table 6:** Confusion matrix for biceps repetitions.

		Actual	
		**Repetition**	**Non-Repetition**
**Detect**	**Repetition**	TRUE Repeat	FALSE Repeat
	**Non-Repetition**	FALSE Non-Repeat	TRUE Non-Repeat

**Table 7:** The performance averages for subject-specific models to detect biceps repetitions throughout the incremental replacement of fatigue repetitions.

			% of Fatigue Repetitions in Dataset					
			0% *	10%	20%	30%	40%	50%
**Models**	**GLM**	Precision	0.94	0.89	0.78	0.71	0.65	0.44
		Recall	0.91	0.81	0.61	0.60	0.52	0.37
		Accuracy	0.90	0.84	0.80	0.76	0.63	0.45
		F1	0.92	0.85	0.68	0.67	0.58	0.40
		%△ F1*	-	−8%	−26%	−30%	−38%	−57%
	**LR**	Precision	0.90	0.85	0.71	0.63	0.49	0.40
		Recall	0.81	0.73	0.53	0.44	0.55	0.36
		Accuracy	0.88	0.83	0.76	0.62	0.57	0.49
		F1	0.85	0.79	0.61	0.52	0.52	0.38
		%△ F1*	-	−9%	−29%	−40%	−41%	−56%
	**RF**	Precision	0.88	0.82	0.68	0.60	0.56	0.45
		Recall	0.78	0.70	0.52	0.50	0.43	0.39
		Accuracy	0.85	0.81	0.75	0.49	0.29	0.19
		F1	0.83	0.76	0.59	0.55	0.49	0.42
		%△ F1*	-	−10%	−30%	−34%	−42%	−50%
	**DT**	Precision	0.86	0.75	0.66	0.57	0.46	0.44
		Recall	0.70	0.64	0.57	0.43	0.40	0.40
		Accuracy	0.81	0.77	0.73	0.61	0.46	0.24
		F1	0.77	0.69	0.61	0.49	0.43	0.42
		%△ F1*	-	−11%	−21%	−36%	−45%	−47%
	**FNN**	Precision	0.98	0.89	0.76	0.68	0.58	0.50
		Recall	0.91	0.79	0.71	0.65	0.60	0.48
		Accuracy	0.99	0.91	0.86	0.80	0.73	0.67
		F1	0.94	0.84	0.73	0.66	0.59	0.49
		%△ F1*	-	−10%	−21%	−31%	−38%	−49%

* Reference to the first set which does not include fatigue. GLM: Generalized Linear Models, LR: Logistic Regression, RF: Random Forest, DT: Decision Tree, and FNN: Feedforward Neural Network.

**Table 8:** The performance averages for cross-subject models to detect biceps repetitions throughout the incremental replacement of fatigue repetitions.

			% of Fatigue Repetitions in Individuals Data					
			0% *	10%	20%	30%	40%	50%
**Models**	**GLM**	Precision	0.85	0.73	0.60	0.52	0.45	0.41
		Recall	0.80	0.71	0.72	0.50	0.32	0.31
		Accuracy	0.87	0.71	0.66	0.52	0.41	0.33
		F1	0.82	0.72	0.65	0.51	0.37	0.35
		%△ F1*	-	−13%	−21%	−38%	−55%	−57%
	**LR**	Precision	0.87	0.79	0.66	0.53	0.45	0.40
		Recall	0.78	0.65	0.58	0.41	0.36	0.30
		Accuracy	0.82	0.75	0.66	0.52	0.43	0.29
		F1	0.82	0.71	0.62	0.46	0.40	0.34
		%△ F1*	-	−13%	−25%	−44%	−51%	−58%
	**RF**	Precision	0.78	0.71	0.64	0.58	0.5	0.46
		Recall	0.67	0.65	0.51	0.45	0.43	0.39
		Accuracy	0.79	0.73	0.58	0.43	0.39	0.21
		F1	0.72	0.68	0.57	0.51	0.46	0.42
		%△ F1*	-	−6%	−21%	−30%	−36%	−41%
	**DT**	Precision	0.78	0.73	0.64	0.53	0.45	0.44
		Recall	0.71	0.63	0.55	0.41	0.41	0.32
		Accuracy	0.81	0.76	0.53	0.42	0.33	0.18
		F1	0.74	0.68	0.59	0.46	0.43	0.37
		%△ F1*	-	−9%	−20%	−38%	−42%	−50%
	**FNN**	Precision	0.90	0.81	0.73	0.66	0.58	0.57
		Recall	0.84	0.75	0.66	0.55	0.48	0.46
		Accuracy	0.95	0.87	0.81	0.74	0.53	0.49
		F1	0.87	0.78	0.69	0.60	0.53	0.51
		%△ F1*	-	−10%	−20%	−31%	−40%	−41%

* Reference to the first set which does not include fatigue.

**Table 9:** The performance averages for subject-specific models to detect biceps repetitions, using the 36 features, throughout the incremental replacement of fatigue repetitions.

			% of Fatigue Repetitions in Dataset					
			0% *	10%	20%	30%	40%	50%
**Models**	**GLM**	Precision	0.96	0.83	0.56	0.49	0.42	0.38
		Recall	0.91	0.81	0.67	0.47	0.30	0.29
		Accuracy	0.99	0.81	0.62	0.49	0.38	0.31
		F$1_{36}$	0.94	0.82	0.61	0.48	0.35	0.33
		%△ F$1_{36}$*	-	−13%	−35%	−49%	−63%	-65%
	**LR**	Precision	0.95	0.86	0.60	0.47	0.37	0.33
		Recall	0.85	0.71	0.53	0.37	0.29	0.24
		Accuracy	0.89	0.82	0.60	0.46	0.35	0.24
		F$1_{36}$	0.90	0.78	0.56	0.41	0.33	0.28
		%△ F$1_{36}$*	-	−13%	−37%	−54%	−64%	−69%
	**RF**	Precision	0.94	0.65	0.59	0.50	0.42	0.38
		Recall	0.80	0.60	0.47	0.39	0.36	0.32
		Accuracy	0.95	0.67	0.54	0.37	0.32	0.17
		F$1_{36}$	0.86	0.62	0.52	0.44	0.38	0.35
		%△ F$1_{36}$*	-	−28%	−39%	−49%	−56%	−59%
	**DT**	Precision	0.90	0.78	0.58	0.42	0.38	0.37
		Recall	0.82	0.67	0.50	0.32	0.35	0.27
		Accuracy	0.93	0.81	0.48	0.33	0.28	0.15
		F$1_{36}$	0.86	0.72	0.54	0.37	0.36	0.31
		%△ F$1_{36}$*	-	−15%	−37%	−57%	−57%	−63%
	**FNN**	Precision	0.99	0.97	0.63	0.57	0.50	0.49
		Recall	0.93	0.90	0.57	0.47	0.41	0.40
		Accuracy	0.99	0.96	0.70	0.64	0.46	0.42
		F$1_{36}$	0.96	0.93	0.60	0.52	0.45	0.44
		%△ F$1_{36}$*	-	−3%	−38%	−46%	−53%	−54%

* Reference to the first set which does not include fatigue.

**Table 10:** The performance averages for cross-subject models to detect biceps repetitions, using the 36 features, throughout the incremental replacement of fatigue repetitions.

			% of Fatigue Repetitions in Dataset					
			0% *	10%	20%	30%	40%	50%
**Models**	**GLM**	Precision	0.92	0.80	0.63	0.58	0.53	0.36
		Recall	0.89	0.73	0.49	0.49	0.42	0.30
		Accuracy	0.88	0.76	0.65	0.62	0.51	0.36
		F$1_{36}$	0.91	0.76	0.56	0.53	0.47	0.33
		%△ F$1_{36}$*	-	−16%	−39%	−42%	−48%	−64%
	**LR**	Precision	0.82	0.76	0.63	0.56	0.40	0.33
		Recall	0.74	0.65	0.47	0.39	0.45	0.29
		Accuracy	0.80	0.74	0.68	0.55	0.46	0.40
		F$1_{36}$	0.78	0.70	0.54	0.46	0.42	0.31
		%△ F$1_{36}$*	-	−10%	−30%	−40%	−46%	−60%
	**RF**	Precision	0.85	0.77	0.59	0.52	0.46	0.37
		Recall	0.76	0.66	0.45	0.44	0.36	0.32
		Accuracy	0.82	0.76	0.65	0.43	0.24	0.16
		F$1_{36}$	0.80	0.71	0.51	0.47	0.40	0.35
		%△ F$1_{36}$*	-	−11%	−36%	−41%	−50%	−57%
	**DT**	Precision	0.91	0.80	0.59	0.45	0.39	0.37
		Recall	0.74	0.69	0.51	0.34	0.34	0.34
		Accuracy	0.85	0.82	0.65	0.48	0.39	0.20
		F$1_{36}$	0.81	0.74	0.55	0.39	0.36	0.36
		%△ F$1_{36}$*	-	−9%	−33%	−52%	−55%	−56%
	**FNN**	Precision	0.99	0.89	0.65	0.58	0.50	0.43
		Recall	0.92	0.79	0.61	0.56	0.52	0.41
		Accuracy	0.99	0.91	0.74	0.69	0.63	0.58
		F$1_{36}$	0.96	0.83	0.63	0.57	0.51	0.42
		%△ F$1_{36}$*	-	−13%	−34%	−40%	−47%	−56%

* Reference to the first set which does not include fatigue.

## Data Availability

The data used in the study is made publicly available at—https://zenodo.org/record/3698242#.XmFZ5qhKguU.
